# Supplementary material for: The role of acceptance and values in quality of life in patients with an acquired brain injury: a questionnaire study
Source: PeerJ. 2017 Jul 6;5:e3545. doi: 10.7717/peerj.3545 (PMC5501966; doi:10.7717/peerj.3545)
Supplement: Supplemental Information 1 [file peerj-05-3545-s002.doc]

Europese hersenletsel vragenlijst

zelfevaluatie

Naam van de patiënt:…………………………………………………………………………

Naam van de verwante:………………………………………………………………………

Datum:………………….

Deze vragenlijst gaat over een aantal problemen of moeilijkheden die mensen soms ervaren in hun leven. Wij zouden graag weten hoe veel u elk van deze hebt ondervonden **gedurende de laatste maand**. Lees aandachtig elke vraag uit de lijst en antwoord door een cirkeltje aan te kruisen in de kolom “helemaal niet” of “een beetje” of “veel”. Sta niet te lang stil bij een vraag. Geef het eerste antwoord dat in je opkomt.

Hoe vaak ondervond u het volgende?

|  | Helemaal niet | Een beetje | Veel |
| --- | --- | --- | --- |
| 1. Hoofdpijn | O | O | O |
| 1. Er niet in slagen dingen op tijd af te krijgen | O | O | O |
| 1. Te snel reageren op wat anderen zeggen of doen | O | O | O |
| 1. Moeilijkheden om dingen te onthouden | O | O | O |
| 1. Moeilijk aan gesprekken kunnen deelnemen | O | O | O |
| 1. Anderen begrijpen je probleem niet | O | O | O |
| 1. Alles kost moeite | O | O | O |
| 1. Niet in staat zijn om activiteiten te plannen | O | O | O |
| 1. Weinig hoop hebben naar je toekomst toe | O | O | O |
| 1. Woede-uitbarstingen hebben | O | O | O |
| 1. Verward zijn | O | O | O |
| 1. Je eenzaam voelen, zelfs in het bijzijn van anderen | O | O | O |
| 1. Stemmingswisselingen hebben, zonder reden | O | O | O |
| 1. Kritisch staan tegenover anderen | O | O | O |
| 1. Taken traag moeten uitvoeren om correct te zijn | O | O | O |
| 1. Flauwvallen of duizelig zijn | O | O | O |
| 1. Gevoelens verbergen voor andere mensen | O | O | O |
| 1. Je droevig voelen | O | O | O |
| 1. Bazig of dominant zijn | O | O | O |
| 1. Herinnerd moeten worden aan de persoonlijke hygiëne | O | O | O |
| 1. Moeilijkheden met het beheer van je geldzaken | O | O | O |
| 1. Problemen met concentratie | O | O | O |
| 1. De gevoelens van anderen niet opmerken | O | O | O |
| 1. Kwaadheid voelen tegenover andere mensen | O | O | O |
| 1. Je gemakkelijk gekwetst voelen | O | O | O |
| 1. Je niet in staat voelen om iets gedaan te krijgen | O | O | O |
| 1. Je geërgerd of geprikkeld voelen | O | O | O |
| 1. Problemen met huishoudelijk karweien | O | O | O |
| 1. Gebrek aan interesse voor hobby’s thuis | O | O | O |
| 1. Je geïsoleerd voelen | O | O | O |
| 1. Je minder waard voelen dan anderen | O | O | O |
| 1. Slaapproblemen | O | O | O |
| 1. Je ongemakkelijk voelen in menigten | O | O | O |
| 1. Als je kwaad bent, schreeuwen tegen andere mensen | O | O | O |
| 1. Moeilijkheden om duidelijk te maken wat je wil zeggen | O | O | O |
| 1. Onzeker zijn over wat te doen in gevaarlijke situaties | O | O | O |
| 1. Koppig zijn | O | O | O |
| 1. Gebrek aan interesse voor uw omgeving | O | O | O |
| 1. Alleen aan jezelf denken | O | O | O |
| 1. Andere mensen wantrouwen | O | O | O |
| 1. Gemakkelijk wenen | O | O | O |
| 1. Moeite om de weg vinden in een nieuwe omgeving | O | O | O |
| 1. Geneigd zijn te veel te eten | O | O | O |
| 1. Vlug in een ruzie verzeild raken | O | O | O |
| 1. Gebrek aan energie hebben of trager reageren | O | O | O |
| 1. De dag van de week vergeten | O | O | O |
| 1. Gevoelens van waardeloosheid hebben | O | O | O |
| 1. Gebrek aan interesse voor hobby’s buitenshuis | O | O | O |
| 1. Hulp nodig hebben bij persoonlijke hygiëne | O | O | O |
| 1. Rusteloosheid | O | O | O |
| 1. Je gespannen voelen | O | O | O |
| 1. Ongepast handelen in gevaarlijke situaties | O | O | O |
| 1. Het gevoel dat het leven niet de moeite waard is | O | O | O |
| 1. Afspraken vergeten | O | O | O |
| 1. Het initiatief aan andere laten in gesprekken | O | O | O |
| 1. Verlies van seksuele interesse of plezier | O | O | O |
| 1. Gooien met voorwerpen als je kwaad bent | O | O | O |
| 1. Liefst alleen zijn | O | O | O |
| 1. Moeilijk beslissingen kunnen nemen | O | O | O |
| 1. Het contact met je vrienden verliezen | O | O | O |
| 1. Gebrek aan interesse voor de lopende zaken | O | O | O |
| 1. Je tactloos gedragen | O | O | O |
| 1. Problemen hebben in het algemeen | O | O | O |

Als één van je naaste verwanten ook deze vragenlijst invult, wil dan a.u.b. de volgende vragen over die persoon beantwoorden.

| 1. Denk je dat het leven van je verwante veranderd is na het oplopen van jouw letsel? | O | O | O |
| --- | --- | --- | --- |
| 1. Denk je dat je verwante problemen heeft door jouw huidige situatie? | O | O | O |
| 1. Denk je dat de stemming van je verwante veranderd is door jouw huidige situatie? | O | O | O |

Nog andere opmerkingen:

…………………………………………………………………………………………………

…………………………………………………………………………………………………

…………………………………………………………………………………………………

…………………………………………………………………………………………………

…………………………………………………………………………………………………

…………………………………………………………………………………………………

Bedankt voor de medewerking.
